# Supplementary material for: A Flower Bud from the Lower Cretaceous of China
Source: Biology (Basel). 2022 Nov 1;11(11):1598. doi: 10.3390/biology11111598 (PMC9687830; doi:10.3390/biology11111598)
Supplement: Supplementary file 1 [file biology-11-01598-s001.zip › biology-1963782-supplementary.pdf]

Supplementary

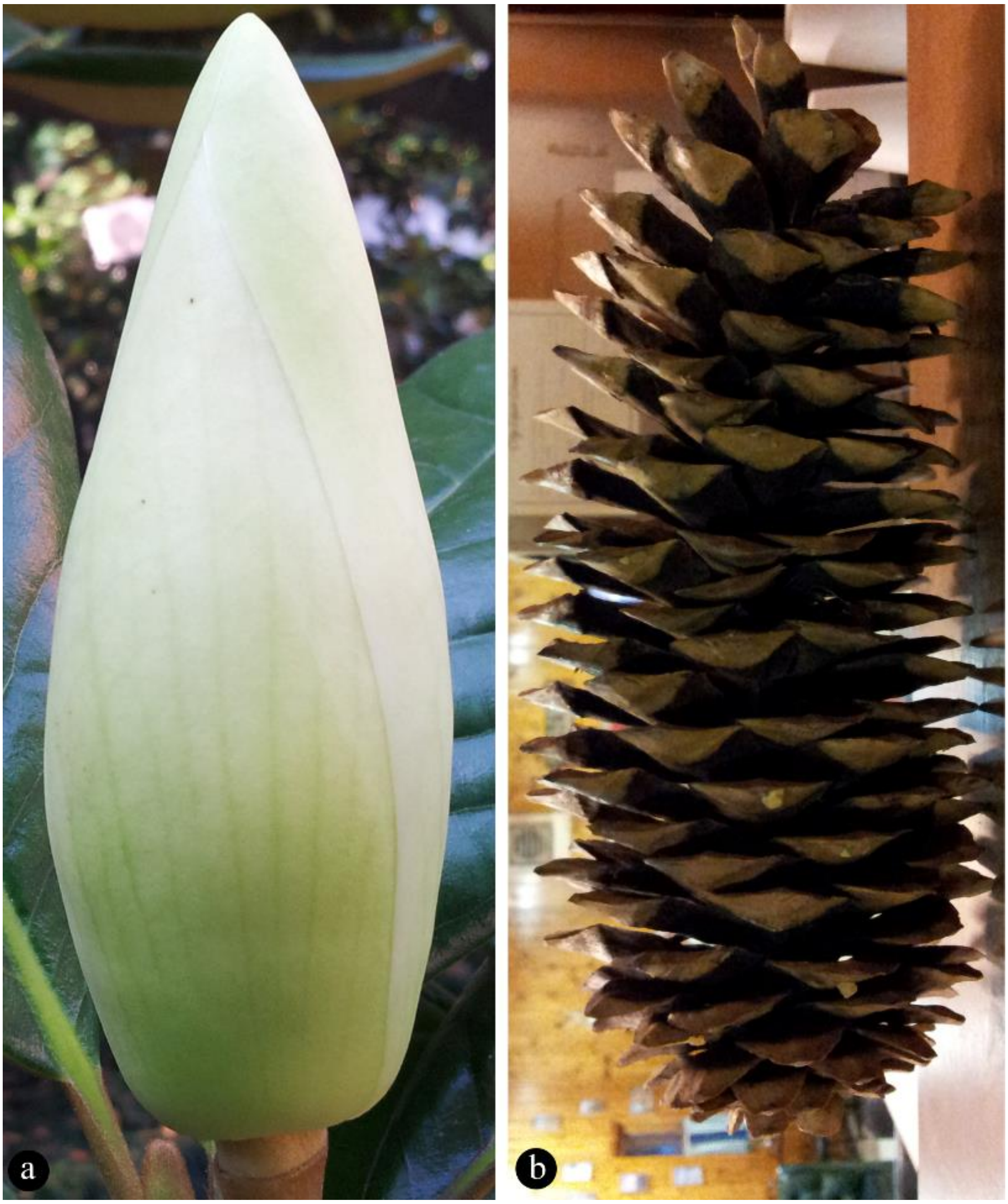

Fig. S1. Comparison between flower bud and coniferous cone. **a.** A flower bud of *Magnolia*, with appressed petals. **b.** A coniferous cone with radially arranged lateral appendages.
